# Serum interleukin-17 A and homocysteine levels in children with autism

**DOI:** 10.1186/s12868-024-00860-5

**Published:** 2024-03-12

**Authors:** Hui Li, Yunhao Dang, Ying Yan

**Affiliations:** 1 Department of Child Health Care, Northwest Women’s and Children’s Hospital, 710061 Xi’an, China; 2 Xi’an Mental Health Center, Department of Children and adolescents Psychology, 710061 Xi’an, China; 3 Department of Child Health Care, Xi’an Central Hospital, 710004 Xi’an, China

**Keywords:** Autism spectrum disorder, Vitamin B12, Folate, Cytokine

## Abstract

**Background:**

Autism Spectrum Disorder (ASD) is a neurodevelopmental condition that typically emerges early in childhood. This study aimed to explore the potential link between serum levels of vitamin B12 and homocysteine (Hcy) and the severity of ASD symptoms in children.

**Methods:**

In this study, 50 children diagnosed with ASD comprised the observation group, while 50 healthy children constituted the control group. Serum levels of IL-17 A, Hcy, folate, and vitamin B12 were compared between the study group and control group, as well as among children with different degrees of ASD severity. The correlation between the Childhood Autism Rating Scale (CARS) score and serum levels of IL-17 A, Hcy, folate, and vitamin B12 was examined. Additionally, the relationship between serum IL-17 A and Hcy levels and their association with the severity ASD were explored.

**Results:**

Compared to the control group, the observation group demonstrated elevated serum Hcy and IL-17 A levels alongside decreased folate and vitamin B12 levels. Individuals with severe ASD exhibited higher Hcy and IL-17 A levels but lower folate and vitamin B12 levels compared to those with mild to moderate ASD. The CARS score showed negative correlations with serum folate and vitamin B12 levels and positive correlations with serum IL-17 A and Hcy levels in ASD patients. Additionally, serum Hcy and IL-17 A levels were correlated with ASD severity.

**Conclusion:**

Children diagnosed with ASD presented with reduced serum vitamin B12 levels and increased levels of Hcy, potentially contributing to the onset and severity of ASD.

**Supplementary Information:**

The online version contains supplementary material available at 10.1186/s12868-024-00860-5.

## Introduction

Autism, also known as Autism Spectrum Disorder (ASD), is the most representative pervasive developmental disorder characterized by impairments in social interaction, communication, and the presence of restricted, stereotyped, and repetitive behaviors. According to estimates from the World Health Organization [1], Approximately 1 in 100 children is affected by autism. ASD exhibits a higher prevalence in males compared to females, with a male-to-female ratio of approximately 4:1 [2]. The etiology of autism is intricately complex, involving various factors such as genetics, environment, and neurodevelopment. Additionally, immune dysregulation, characterized by abnormal immune system activity, plays a crucial role in the pathogenesis of autism. Evidence of immune dysfunction is frequently observed in individuals with ASD, often associated with behavioral deterioration [3]. Folate and vitamin B12 play critical roles in one-carbon metabolism, and perturbations in their metabolism have been observed in numerous individuals with Autism Spectrum Disorder (ASD), implying a potential involvement of the folate-tryptophan cycle in autism pathogenesis [4].. Disruption of biochemical pathways associated with folate and vitamin B12 is linked to the onset and severity of autism. These pathways, including methylation and transsulfuration, are involved in neurodevelopmental processes. Dysregulation of these pathways may result in imbalances that could potentially impact the symptoms of autism [5, 6].. Genetic factors play a substantial role in autism, as evidenced by elevated concordance rates of ASD in monozygotic twins, as well as an increased risk in families with affected individuals. Autism involves numerous genes, including those associated with synaptic function, neuronal signaling, and chromatin remodeling [7]. However, the genetic architecture of autism is highly heterogeneous, with different combinations of genetic variants contributing to individual cases. Environmental variables may also contribute to the pathogenesis of autism. Prenatal and postnatal factors such as maternal drug use, infections, and immunological activation have all been linked to an increased risk of ASD [8]. Additionally, environmental toxins, such as air pollutants and certain chemicals, may impact the development of autism [9].

Individuals with autism often exhibit signs of immune dysregulation and heightened immune activation [10]. Increased levels of pro-inflammatory cytokines, such as interleukin-17 A (IL-17 A), tumor necrosis factor-alpha (TNF-alpha), and interleukin-1 beta (IL-1β), have been observed in the blood and cerebrospinal fluid of individuals with autism [11–14]. Furthermore, maternal immune activation during pregnancy, characterized by an exaggerated immune response and heightened production of pro-inflammatory factors, has been suggested as a potential risk factor for autism in offspring [11].. These pro-inflammatory factors participate in diverse inflammatory pathways and can negatively impact neuronal function and development. The involvement of inflammation in autism is complex and multifactorial, with immune dysregulation and heightened inflammation hypothesized to potentially contribute to the development and progression of autism symptoms. Previous research suggests that children with ASD may exhibit higher food selectivity compared to control groups, often leading to nutritional deficiencies [15, 16]. Bandini and other researchers have reported that children with Autism Spectrum Disorder (ASD) exhibit increased aversive eating behaviors [16], leading to a limited variety of food consumption, which may contribute to nutritional deficiencies. However, there remains a necessity for comprehensive analyses encompassing all facets of nutritional intake in children with ASD. This includes examining the frequency of food consumption across different groups, assessing the extent of deficiencies in individuals with ASD, and exploring their dietary preferences. Additionally, aside from inflammatory factors, elevated blood homocysteine levels have been linked to various neurological and mental disorders, including autism [17, 18].. Homocysteine is an amino acid involved in methylation reactions and the metabolism of folate and vitamin B12 [19]. It can have neurotoxic effects, impacting neuronal function, neurotransmitter synthesis, and oxidative stress levels [20]. Additionally, homocysteine can disrupt DNA methylation, an essential epigenetic mechanism that regulates gene expression during brain development and function. High levels of homocysteine are associated with alterations in neurotransmitter systems, such as glutamate and gamma-aminobutyric acid (GABA), both crucial for brain function and potentially contributing to the core symptoms of autism [21]. While the association between homocysteine and autism is still being investigated, evidence suggests that reducing elevated homocysteine levels through nutritional interventions, such as folate, vitamin B12, and vitamin B6 supplementation, may have beneficial effects on behavioral and cognitive symptoms in certain individuals with autism [22, 23].

The aim of this study was to evaluate serum levels of interleukin-17 A (IL-17 A) and homocysteine (Hcy) in a cohort of children diagnosed with autism spectrum disorder (ASD) and examine the potential correlation between these serum biomarkers and the severity of ASD symptoms.

## Materials and methods

### Participants

Fifty individuals diagnosed with ASD were recruited for this study at the Center for Autism of Northwest Women’s and Children’s Hospital between September 2021 and June 2023. The Diagnosis was based on the criteria outlined in the Diagnostic and Statistical Manual of Mental Disorders, Fifth Edition (DSM-V) [24]. To ensure a comprehensive assessment of the clinical profile, in addition to DSM-V, the Autism Diagnostic Interview-Revised (ADI-R) and the Autism Diagnostic Observation Schedule (ADOS) were employed.

The eligible participants were children aged 3 to 12 years who had received a diagnosis of ASD, and whose parents provided consent for their participation in the research. Exclusions from the study comprised children with chronic diseases, infections, and other physical or neurological abnormalities. Also excluded were those currently taking medications, supplements, or vitamins.

#### Vitamin B12 deficiency

We also assessed the participants for vitamin B12 levels to explore potential associations with ASD. Blood samples were collected. Any participants found to have vitamin B12 deficiency were documented and considered in the analysis.

#### Gastrointestinal symptoms

Comprehensive medical histories, including the presence of gastrointestinal symptoms, were obtained from the participants and their parents or caregivers. This information was crucial in understanding the potential impact of gastrointestinal symptoms on the serum levels of analyzed parameters, such as folate and vitamin B12.

To provide a control group, 50 healthy candidates, matched for age and sex, were enrolled as volunteers with the permission of their parents.

The research was conceptualized and conducted in accordance with the principles outlined in the Helsinki Declaration. All participants were recruited within the same timeframe, and informed consent forms were completed by the primary caregivers. Ethical approval for this study was obtained from the ethics committee at Northwest Women’s and Children’s Hospital.

### Clinical evaluation

The assessment of individuals with ASD involved a comprehensive review of the clinical record provided by the caregivers, a clinical examination, and a neuropsychiatric evaluation. The severity of ASD was quantified using the Childhood Autism Rating Scale (CARS) [25], which evaluates a child across fifteen domains on a scale of one to hour. Children scoring between 30 and36 on the scale were classified as having mild to moderate ASD, while those with values ranging from 37 to 60 points were categorized as having severe ASD.

### Blood sampling

Collect 2 milliliters of venous blood sample into serum separation tubes and centrifuge immediately at 1500 g for 10 min. Store the serum samples at -80 °C until testing. Use human folate ELISA assay kit (Roche, cat. no. RAB0659 sensitivity 0.1 ng/mL, assay range 0.102-25 ng/mL), human homocysteine ELISA kit (Roche, cat. no. EY-01H807, sensitivity 0.5 ng/mL, assay range 0.5 ng/mL to 50 ng/mL), and Thermofisher human interleukin 17a (Hu IL-17 A) ELISA kit (cat. no. 88-7176-88, sensitivity 4 pg/mL, assay range 4-500 pg/mL), following the manufacturer’s instructions.

### Statistics analysis

Graphpad 8.4 and Rstudio software were used. Continuous data were reported as mean standard deviation, whilst categorical data were presented as percentages.

The continuous variables were initially assessed for normal distribution using the Kolmogorov-Smirnov test. If both sets of data followed a normal distribution, the Student’s t-test was applied. In cases where non-normal distribution was observed, the Mann-Whitney U test was utilized. The simultaneous use of Levene’s test is employed to assess the homogeneity of variances for continuous variables. For quantitative data intergroup comparisons, the Chi-square test or Fisher’s exact Chi-square test was utilized. To evaluate the association between two measured values in the groups, Pearson’s and Spearman correlation tests were utilized.

## Results

### Characteristics of participants

The demographic and clinical characteristics of the study participants, along with the severity of autism spectrum disorder (ASD) as measured by the Childhood Autism Rating Scale (CARS) total score, are presented in Table 1. The mean age of all participants was 4.22 years (SD = 0.39). Children in the ASD group had a slightly higher mean age of 4.25 years (SD = 0.31) compared to the control group’s mean age of 4.19 years (SD = 0.45), although these differences were not statistically significant (*p* > 0.05). Additionally, there were no significant differences in age, gender distribution, or body mass index (BMI) between children with ASD and healthy subjects (*p* > 0.05). Among children with ASD, 54% exhibited mild to moderate symptoms, while the remaining 46% displayed severe symptoms. Detailed results of normality and homogeneity tests for all data are provided in Supplementary Table 1.

**Table 1:** Demographic and clinical characteristic of patients and healthy controls

Characteristics	Overall (*N* = 100)	ASD (*N* = 50)	Control (*N* = 50)	*p*-value^1^
Age, years	4.22 ± 0.39	4.25 ± 0.31	4.19 ± 0.45	0.4
Gender, male n (%)	58 (58.0)	27 (54.0)	31 (62.0)	0.4
BMI, kg/m^2^	16.53 ± 1.58	16.70 ± 1.26	16.37 ± 1.85	0.2
CARS total score		37 ± 2	0	-
Severity of ASD				
Mild to moderate n (%)	27 (27.0)	27 (54.0)	0 (0.0)	-
Severe n (%)	23 (23.0)	23 (46.0)	0 (0.0)	-
Picky eating, n (%)	49 (49.0)	39 (78.0)	10 (20.0)	< 0.001
Resistance to vegetables, n (%)	50 (50.0)	41 (82.0)	9 (18.0)	< 0.001
Resistance to meats, n (%)	27(27.0)	15 (30.0)	12 (24.0)	0.653
Resistance to fruits, n (%)	17(17.0)	7 (14.0)	10 (20.0)	0.595

^*1*^ Wilcoxon rank sum test; Pearson’s Chi-squared test

### Comparison of serum cytokine, homocysteine, folate and vitamin B12 levels between children with ASD and control groups

**Fig. 1:**
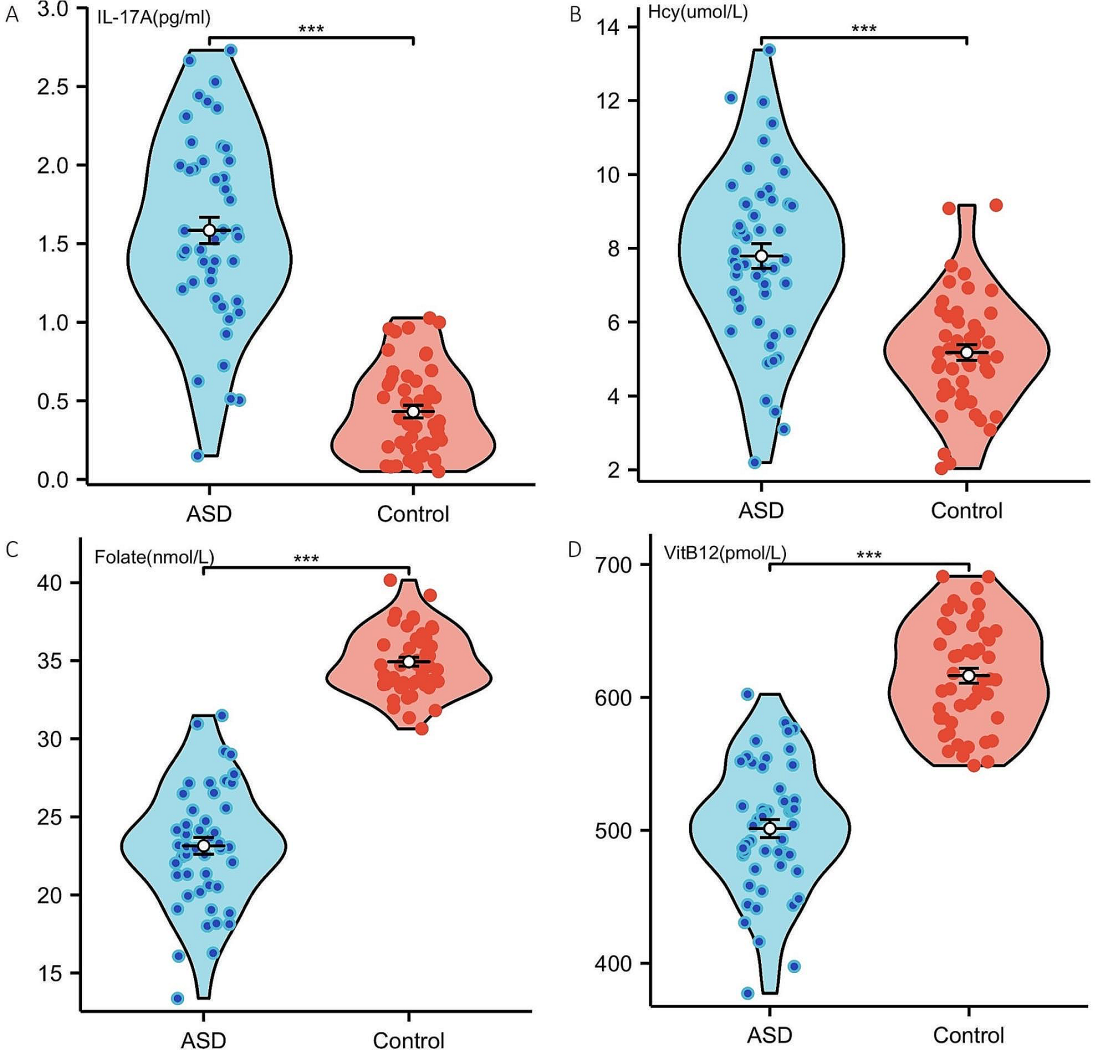
IL-17 A, Hcy, Folate, and VitB12 were compared in the ASD and control groups. *** indicates a *P*-value less than 0.001

In Table 2, a comparative analysis of serum IL-17 A, homocysteine (Hcy), folate, and vitamin B12 levels among individuals with varying degrees of autism spectrum disorder (ASD) (mild, moderate, or severe) and age- and sex-matched control children is presented. In the ASD group, IL-17 A levels were significantly elevated (1.48 pg/ml) compared to the control group (0.44 pg/ml) with a *p*-value of less than 0.001. Vitamin B12 concentrations were notably reduced in children with ASD (502.69 pmol/L) compared to controls (626.41 pmol/L), demonstrating a statistically significant difference with a *p*-value less than 0.001. In comparison to the control group, the ASD group exhibited significantly elevated levels of IL-17 A and Hcy. Conversely, blood concentrations of folate and vitamin B12 were 1.6-fold and 1.2-fold higher in healthy children compared to those with ASD, respectively (Fig. 1 A, B, C and D). In the cohort of 50 ASD patients, 7 presented with gastrointestinal symptoms. There was no statistically significant difference in the levels of B12 between the two groups, those with and without gastrointestinal symptoms (see Supplementary Table 2).

**Table 2:** Laboratory results of patients and healthy controls

Variables	ASD	mASD	sASD	Control	*p*-value^1^	*p*-value^2^	*p*-value^3^
IL-17 A, pg/ml	1.48 ± 0.60	1.32 ± 0.57	1.68 ± 0.59	0.44 ± 0.61	< 0.001	< 0.001	< 0.001
Hcy, μmol/L	7.79 ± 2.24	7.29 ± 1.26	8.37 ± 2.90	4.84 ± 1.39	< 0.001	< 0.001	< 0.001
Folate, nmol/L	22.32 ± 3.64	23.94 ± 1.98	20.94 ± 4.13	35.09 ± 2.46	< 0.001	< 0.001	< 0.001
VitB12, pmol/L	502.69 ± 46.26	525.19 ± 41.58	476.28 ± 36.50	626.41 ± 46.64	< 0.001	< 0.001	< 0.001

Abbreviation: mASD: mild Autism Spectrum Disorder; sASD: severe Autism Spectrum Disorder.1, ASD vs. Control; 2, mASD vs. Control; 3, sASD vs. Control

### Associations between serum IL-17 A and homocysteine levels with CARS total scores in the ASD group

An analysis of the association between blood levels of IL-17 A, Hcy, folate, and vitamin B12 with the Childhood Autism Rating Scale (CARS) total score in individuals with autism spectrum disorder (ASD) reveals significant findings. Higher IL-17 A levels (*r* = 0.65) and elevated homocysteine levels (*r* = 0.62) positively correlated with increased severity of ASD. Conversely, lower folate levels (*r* = -0.90) and reduced vitamin B12 levels (*r* = -0.79) negatively correlated with higher severity of ASD symptoms. These numerical associations provide insights into potential biomarkers for ASD (Table 3).

**Table 3:** Correlations between serum IL-17 A and homocysteine levels with the CARS total scores in the ASD group

Variables	CARS total score	
	Correlation (r)	*p*-value
IL-17 A, pg/ml	0.65	< 0.001
Hcy, μmol/L	0.62	< 0.001
Folate	-0.90	< 0.001
VitB12	-0.79	< 0.001

### Associations of serum IL-17 A and homocysteine levels and their interaction with ASD severity

The association between serum IL-17 A and homocysteine (HCY) levels and the severity of Autism Spectrum Disorder (ASD) is categorized into mild-to-moderate symptoms (MASD) and severe symptoms (SASD) (Table 4). The results indicate a strong positive correlation (*r* = 0.68) between IL-17 A levels and ASD severity, with mASD showing 1.32 pg/ml (± 0.57) and sASD exhibiting 1.68 pg/ml (± 0.59). Similarly, Hcy levels also displayed a positive correlation (*r* = 0.63) with ASD severity, where mASD had 7.29 μmol/L (± 1.26) and sASD showed 8.37 μmol/L (± 2.90). Both correlations were statistically significant (*p* < 0.001), emphasizing the potential role of IL-17 A and Hcy in influencing the severity of ASD symptoms. Subgroup analysis was conducted based on gender for ASD patients and the control group. No statistically significant differences were observed in the levels of IL-17 A, Hcy, Folate, and VitB12 among ASD patients of different genders. Refer to Supplementary Table 3 for details.

**Table 4:** Associations of the serum IL-17 A and Hcy levels with ASD severity

Variables	mASD	sASD	r	*p*-value
IL-17 A, pg/ml	1.32 ± 0.57	1.68 ± 0.59	0.68	< 0.001
Hcy, μmol/L	7.29 ± 1.26	8.37 ± 2.90	0.63	< 0.001

Abbreviation: mASD: mild Autism Spectrum Disorder; sASD: severe Autism Spectrum Disorder

## Discussion

ASD is a neurodevelopmental disorder characterized by diverse clinical manifestations, including repetitive and stereotyped behaviors, impaired verbal communication skills, and deficits in social interaction [2]. The prevalence of ASD has been steadily increasing, highlighting the importance of understanding its underlying biological mechanisms and identifying potential biomarkers for diagnostic and therapeutic purposes. This study aimed to explore the association between blood levels of homocysteine (Hcy) and IL-17 A and the severity of ASD in children. Hcy, an amino acid containing sulfur, exerts cytotoxic effects by inducing apoptosis and disrupting normal cellular processes during embryonic development [26, 27]. Under physiological conditions, Hcy is maintained in dynamic equilibrium within the body. However, impairments in its metabolic pathways can lead to its accumulation, resulting in a condition called hyperhomocysteinemia [28]. Hcy is primarily metabolized through two pathways: the methylation pathway and the transsulfuration pathway [29]. The methylation pathway involves the utilization of vitamin B12 as a coenzyme and the formation of S-adenosylmethionine through the action of S-adenosylmethionine synthetase [30]. The transsulfuration pathway, catalyzed by cystathionine synthetase, is an alternate pathway for homocysteine (Hcy) metabolism. Elevated levels of Hcy can lead to the generation of reactive oxygen species and hydrogen peroxide, resulting in the inhibition of antioxidant enzymes and exacerbation of lipid peroxidation [31]. Our study observed higher levels of Hcy in children with ASD, suggesting an association with increased oxidative stress in ASD. B vitamins, including vitamin B6, vitamin B12, and folic acid, play essential roles in neurodevelopment. Deficiencies in these vitamins have been linked to an elevated risk of neurological diseases, neurodevelopmental disorders, and Alzheimer’s disease [32, 33]. As coenzymes, vitamin B12 promotes protein and nucleic acid biosynthesis and enhances the utilization of folic acid, thereby offering protection to the nervous system [34]. Findings from our study demonstrated folate and vitamin B12 levels were significantly lower in children with ASD compared to the healthy control. Similar to Li et al [35]., who compared multiple research groups and found that 5-HT, feeding practices, Hcy, vitamin B12 levels, and febrile seizures are major risk factors for autism in children, with significant correlations. However, there are conflicting findings as well. Wen-Xiong Chen et al [36]., in their study analyzing the plasma amino acid profile of 110 cases of autism spectrum disorder children in southern China, reported a decrease in Hcy levels in the plasma of ASD patients. These findings suggest an imbalance in the one-carbon metabolism pathway, which is involved in methylation reactions and essential for normal neurological development.

Research has consistently indicated that specific cytokines, produced by both immune and non-immune cells upon activation, can have detrimental effects on neurodevelopment and behavioral outcomes [37, 38]. Perturbations in cytokine levels have been observed in response to infections, diseases, and exposure to various toxic substances, suggesting that abnormal cytokine levels could result from environmental factors influencing autism [39, 40]. Several studies have reported alterations in cytokine profiles in children with ASD.

Alterations in cytokine profiles in children with ASD and notable changes in cytokines and their receptors are highlighted. Key cytokines and their receptors investigated in relation to childhood autism include IL-17 A, IL-1β, IL-2R, IL-4, IL-6, and various interleukins, each with diverse cellular origins and target cell types [41, 42]. Consistent with previous studies, our results revealed elevated levels of IL-17 A in children with ASD, indicative of increased inflammation associated with ASD.

In the ASD cohort, we observed a correlation between the severity of Autism Spectrum Disorder (ASD), as assessed by the Childhood Autism Rating Scale (CARS) score, and serum levels of IL-17 A, homocysteine (Hcy), folate, and vitamin B12. Higher CARS scores were specifically associated with elevated levels of IL-17 A and Hcy, while showing reduced levels of folate and vitamin B12. The CARS, developed by Schopler in the 1980s, serves as a widely used tool for screening and diagnosing autism [25]. Within our study, 23 out of the 50 children with severe ASD and 27 out of the 50 children with mild-to-moderate ASD were included based on the CARS criteria. Our findings revealed heightened serum IL-17 A and Hcy levels and decreased folate and vitamin B12 levels in children with severe ASD compared to those with mild-to-moderate ASD. Furthermore, correlation analyses unveiled significant negative associations between CARS scores and serum vitamin B12 levels, as well as significant positive associations between CARS scores and serum Hcy levels among children with ASD. The negative correlation between CARS scores and serum folate and vitamin B12 levels is consistent with previous studies demonstrating the importance of B vitamins in neurodevelopment. Deficiencies in these vitamins have been associated with an increased risk of neurological disorders. Furthermore, our study emphasizes the connection between IL-17 A, a pro-inflammatory cytokine, and the severity of ASD. Dysregulated cytokine levels, indicative of immune system dysfunction and potential neuroinflammation, have been reported in individuals with ASD [43]. Elevated IL-17 A levels in children with ASD provide additional support for the involvement of inflammatory processes in the pathogenesis of ASD. The increase in IL-17 A has been demonstrated to reflect the methylation of its mRNA. In the study by Wang et al [44]., an analysis of the activity of a reporter gene derived from pGL3 containing IL-17 A mRNA fragments revealed that the methylation by NSun2 promotes the translation of IL-17 A. In summary, NSun2 mediates the upregulation of IL-17 A expression induced by HHcy by methylating IL-17 A mRNA and facilitating its translation in T lymphocytes. Abdullah et al. also observed an increase in anti-inflammatory markers in the ASD patient group [45].

Additionally, our study acknowledges the prevalence of picky eating and resistance to consuming vegetables among individuals with ASD, which could contribute to low levels of B12 and folate. Previous research suggests that individuals with ASD may have heightened sensitivity to the taste, texture, and color of food, which could lead to aversion towards certain foods, such as vegetables. This aversion to vegetables, often accompanied by a preference for processed or specific food items, may result in a diet lacking in essential vitamins and minerals. Furthermore, individuals with ASD may experience sensory integration difficulties, affecting their perception and processing of external stimuli. The discomfort triggered by the texture and mouthfeel of vegetables may exacerbate their resistance to eating them, potentially contributing to nutritional deficiencies, including low B12 and folate levels [46].

This study has some limitations. Firstly, it is a single-center retrospective study, and future research would benefit from a larger sample size to enhance the validation of our conclusions. Secondly, the primary methodology employed in this study was ELISA. However, we acknowledge the importance of utilizing supplementary techniques for further validation. In subsequent research, we plan to incorporate Western blotting or flow cytometry to strengthen the robustness of our study findings. These considerations aim to address the current limitations and improve the overall reliability and generalizability of our research outcomes.

In summary, our investigation yields compelling evidence of disrupted serum levels of vitamin B12, homocysteine (Hcy), IL-17 A, and folate in children diagnosed with Autism Spectrum Disorder (ASD), suggesting potential implications in both the initiation and severity of the condition. These outcomes underscore the critical need for expanded research into the intricate connections between immune dysregulation, one-carbon metabolism, and neurodevelopment in the context of ASD.

### Electronic supplementary material

Below is the link to the electronic supplementary material.

Supplementary Material 1

Supplementary Material 2

Supplementary Material 3

## Data Availability

No datasets were generated or analysed during the current study.
