# Supplementary material for: Serum interleukin-17 A and homocysteine levels in children with autism
Source: BMC Neurosci. 2024 Mar 12;25:17. doi: 10.1186/s12868-024-00860-5 (PMC10935804; doi:10.1186/s12868-024-00860-5)
Supplement: Supplementary file 1 — Supplementary Material 1 [file 12868_2024_860_MOESM1_ESM.docx]

Supplementary Table 1. Normality and Homogeneity Tests for the ASD Group and Control Group.

| **Variables** | **ASD** | **Control** | | | |  |
| --- | --- | --- | --- | --- | --- | --- |
|  | **Kolmogorov-Smirnov** | **Kolmogorov-Smirnov** | | **Levene's** | |  |
| **Age, years** | 0.200 |  | 0.200 | | 0.053 | |
| **BMI, kg/m2** | 0.200 |  | 0.200 | | 0.099 | |
| **IL-17A, pg/ml** | 0.200 |  | 0.200 | | 0.468 | |
| **Hcy, μmol/L** | 0.200 |  | 0.200 | | 0.116 | |
| **Folate, nmol/L** | 0.200 |  | 0.200 | | 0.278 | |
| **VitB12, pmol/L** | 0.200 |  | 0.200 | | 0.312 | |

The results of both the Kolmogorov-Smirnov (K-S) test and Levene's test yielded significant p-values
