# Supplementary material for: Serum interleukin-17 A and homocysteine levels in children with autism
Source: BMC Neurosci. 2024 Mar 12;25:17. doi: 10.1186/s12868-024-00860-5 (PMC10935804; doi:10.1186/s12868-024-00860-5)
Supplement: Supplementary file 2 — Supplementary Material 2 [file 12868_2024_860_MOESM2_ESM.docx]

Supplementary Table 2. Vitamin B12 Levels in ASD Patients with Gastrointestinal Symptoms.

| **Variables** | **Gastrointestinal Symptoms**, N = 7 | **No-GS**, N = 43 | ***P*-value** |
| --- | --- | --- | --- |
| **VitB12, pmol/L** | 498.57(47.88) | 504.18 (44.23) | 0.759 |
