# Supplementary material for: Serum interleukin-17 A and homocysteine levels in children with autism
Source: BMC Neurosci. 2024 Mar 12;25:17. doi: 10.1186/s12868-024-00860-5 (PMC10935804; doi:10.1186/s12868-024-00860-5)
Supplement: Supplementary file 3 — Supplementary Material 3 [file 12868_2024_860_MOESM3_ESM.docx]

Supplementary Table 3. Comparison between the ASD group and the Control group based on gender differences.

| **Variables** | **ASD** | |  | **Control** | |  | |  |  |
| --- | --- | --- | --- | --- | --- | --- | --- | --- | --- |
|  | **Male** | **Female** | **P-value** | **Male** | **Female** | | **P-value** | |  |
| **Age, years** | 4.21±0.28 | 4.28±0.35 | 0.436 | 4.15±0.41 | 4.22±0.52 | | 0.599 | | |
| **BMI, kg/m2** | 17.81±1.32 | 15.22±1.21 | <0.001 | 17.45±1.77 | 15.64±1.90 | | <0.001 | | |
| **IL-17A, pg/ml** | 1.44±0.57 | 1.53±0.63 | 0.766 | 0.51±0.58 | 0.37±0.64 | | 0.430 | | |
| **Hcy, μmol/L** | 7.54±2.48 | 7.92±2.18 | 0.571 | 4.55±1.48 | 5.10±1.32 | | 0.191 | | |
| **Folate, nmol/L** | 22.18±3.78 | 22.49±3.57 | 0.768 | 33.71±2.31 | 36.78±2.67 | | <0.001 | | |
| **VitB12, pmol/L** | 498.77±45.84 | 507.21±47.22 | 0.525 | 615.88±44.38 | 635.12±48.15 | | 0.156 | | |
